# Longitudinal profiling in patients undergoing cardiac surgery reveals postoperative changes in DNA methylation

**DOI:** 10.1186/s13148-022-01414-4

**Published:** 2022-12-30

**Authors:** Matthew A. Fischer, Douglas J. Chapski, Elizabeth Soehalim, Dennis J. Montoya, Tristan Grogan, Matteo Pellegrini, Hua Cai, Richard J. Shemin, Thomas M. Vondriska

**Affiliations:** 1 Department of Anesthesiology & Perioperative Medicine, David Geffen School of Medicine, UCLA, CHS 37-100, 650 Charles Young Dr, Los Angeles, CA 90095 USA; 2 Department of Molecular, Cellular & Developmental Biology, David Geffen School of Medicine, UCLA, Los Angeles, USA; 3 Department of Surgery, David Geffen School of Medicine, UCLA, Los Angeles, USA; 4 Department of Medicine, David Geffen School of Medicine, UCLA, Los Angeles, USA; 5 Department of Physiology, David Geffen School of Medicine, UCLA, Los Angeles, USA

**Keywords:** DNA methylation, Cardiac surgery, Precision medicine, Atrial fibrillation

## Abstract

**Background:**

Cardiac surgery and cardiopulmonary bypass induce a substantial immune and inflammatory response, the overactivation of which is associated with significant pulmonary, cardiovascular, and neurologic complications. Commensurate with the immune and inflammatory response are changes in the heart and vasculature itself, which together drive postoperative complications through mechanisms that are poorly understood. Longitudinal DNA methylation profiling has the potential to identify changes in gene regulatory mechanisms that are secondary to surgery and to identify molecular processes that predict and/or cause postoperative complications. In this study, we measure DNA methylation in preoperative and postoperative whole blood samples from 96 patients undergoing cardiac surgery on cardiopulmonary bypass.

**Results:**

While the vast majority of DNA methylation is unchanged by surgery after accounting for changes in cell-type composition, we identify several loci with statistically significant postoperative changes in methylation. Additionally, two of these loci are associated with new-onset postoperative atrial fibrillation, a significant complication after cardiac surgery. Paired statistical analysis, use of FACS data to support sufficient control of cell-type heterogeneity, and measurement of IL6 levels in a subset of patients add rigor to this analysis, allowing us to distinguish cell-type variability from actual changes in methylation.

**Conclusions:**

This study identifies significant changes in DNA methylation that occur immediately after cardiac surgery and demonstrates that these acute alterations in DNA methylation have the granularity to identify processes associated with major postoperative complications. This research also establishes methods for controlling for cell-type variability in a large human cohort that may be useful to deploy in other longitudinal studies of epigenetic marks in the setting of acute and chronic disease.

**Supplementary Information:**

The online version contains supplementary material available at 10.1186/s13148-022-01414-4.

## Background

With approximately 300,000 procedures performed annually in the USA, cardiac surgeries are common invasive procedures that often require a cardiopulmonary bypass to provide a bloodless field during the operation [1]. Cardiac surgery with cardiopulmonary bypass induces a major immunologic and inflammatory response [2–4] secondary to ischemia, reperfusion injury, surgical trauma, and contact system activation during surgery [3]. This activated inflammatory response contributes to the occurrence of acute pulmonary, cardiovascular, neurologic, and hematologic dysfunction after cardiac surgery [2]. The release of immune mediators is significantly augmented with cardiopulmonary bypass compared to cardiac surgery without bypass [5].

In the postoperative period, the immune system works to promote healing and to fight infection [6]. Although the immune and inflammatory response to cardiac surgery has been an active area of clinical and basic research, the mechanisms driving associated complications in the postoperative setting are insufficiently understood [2, 7, 8], precluding therapeutic intervention. Epigenetic regulation of the immune response has been examined in colorectal and hip surgery [9] but has not been studied in cardiac surgery.

DNA methylation is a dynamic epigenetic modification that can alter gene expression [10]. In this study, we focus on the covalent attachment of a methyl group at the fifth carbon of cytosine residues (CpGs) in DNA, which has been associated with gene silencing (when in the promoter or transcription start site) or increased expression (when in the gene body), depending on the location of the methylated cytosine relative to the gene [10]. Previous investigations have shown that acute changes in DNA methylation can identify mechanistic responses in peripheral leukocytes [9, 11] following the profound degree of inflammation in major surgery (colorectal and hip replacement), and overactivation of these inflammatory pathways is known to be associated with significant postoperative complications. Immunomodulation strategies such as corticosteroids have been studied in response to cardiopulmonary bypass but remain controversial given that these blunt tools have significant side effects [2], such as hyperglycemia and immunosuppression. Currently, there is an unmet need in understanding the regulation of the immune response to surgery, particularly the separation of adaptive immune responses to those that are associated with postoperative complications.

In this study, we enrolled 96 patients undergoing cardiac surgery on cardiopulmonary bypass and measure DNA methylation in whole blood samples preoperatively and again at 24 h postoperatively. We establish methods for longitudinal monitoring of epigenetic changes in live patients and demonstrate controlling for immune cell heterogeneity. We identify DNA methylation loci with statistically significant postoperative changes after cardiac surgery on cardiopulmonary bypass, two of which are significantly associated with postoperative atrial fibrillation (POAF), a major postoperative complication associated with increased morbidity and mortality [12]. We reason that the identification of specific mechanisms within the immune response to surgery that contributes to adverse outcomes will yield targets for modulating this response to reduce significant postoperative complications.

## Results

**Fig. 1:**
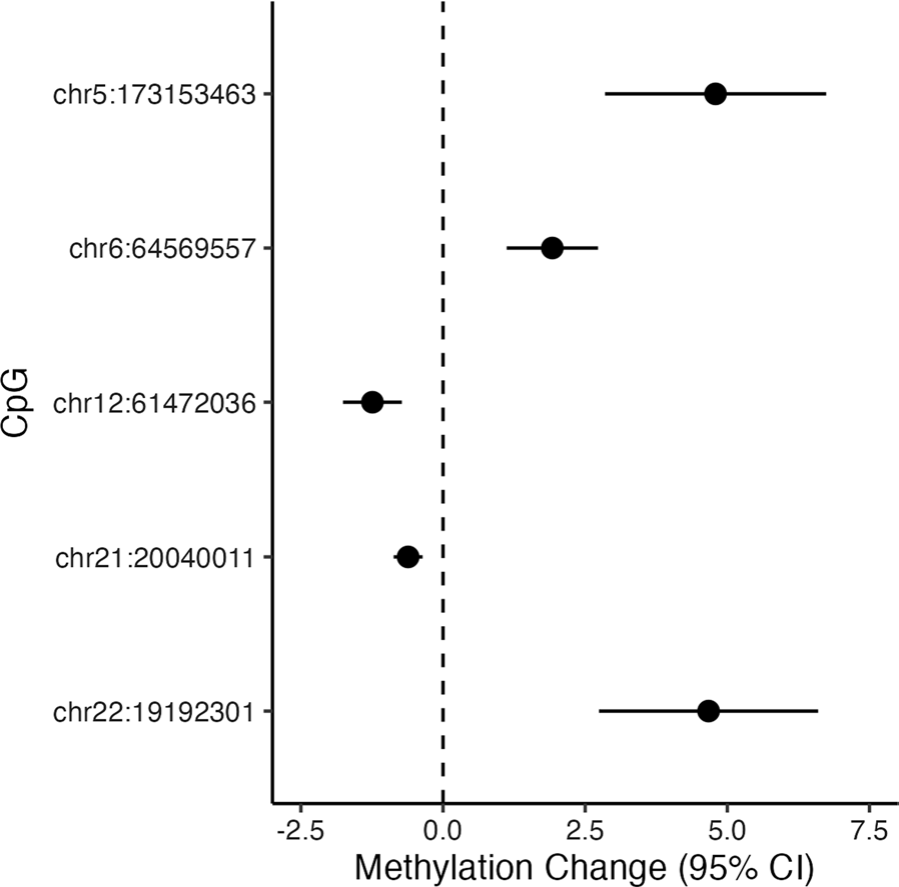
Mean methylation change and 95% CI for CpGs with a statistically significant change after cardiac surgery. For each significant CpG, the mean and 95% confidence interval (CI) of the methylation change after cardiac surgery are shown

**Fig. 2:**
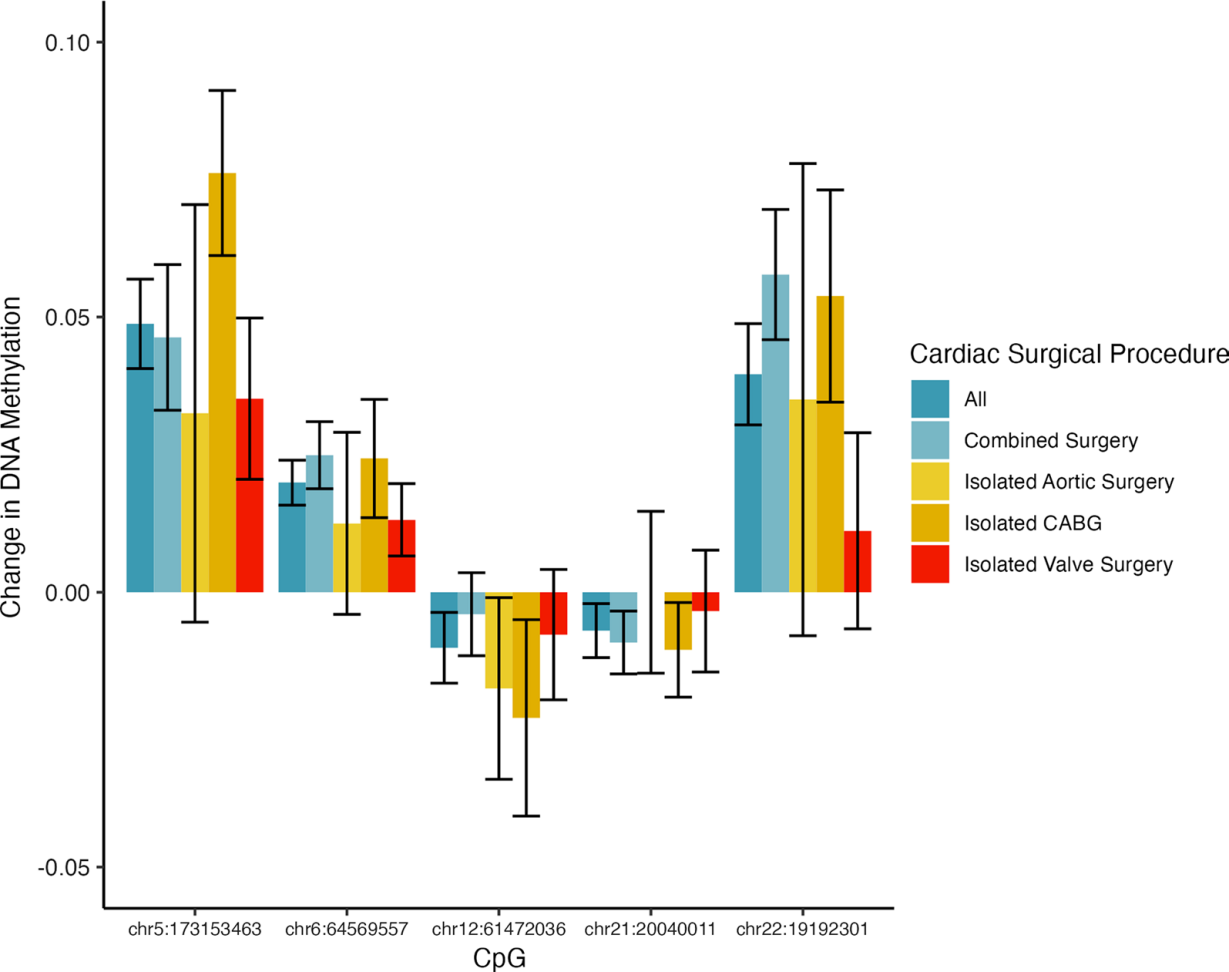
CpGs with a statistically significant change in DNA methylation after cardiac surgery: Methylation change by cardiac surgical procedure. Mean changes in DNA methylation with standard error bars are shown for each CpG that has a statistically significant change in DNA methylation after cardiac surgery by type of cardiac surgical procedure. Combined surgeries are those with two or more of the following surgical procedures: aortic surgery, coronary artery bypass grafting (CABG) surgery, myomectomy and valve surgery

The characteristics of the enrolled patient population are given in Table 1. There were 40,343 CpGs with 10X coverage measured in all preoperative and postoperative samples in all patients. An important initial observation, with general implications for studies of this nature, is that there are 6587 sites that are significantly different between preoperative and postoperative samples (FDR < 0.05) before controlling for cell-type estimates, highlighting the importance of accounting for cell-type heterogeneity. After controlling for cell-type estimates in preoperative and postoperative samples, we identified 5 CpGs (Table 2) that have statistically significant changes (FDR < 0.05) in DNA methylation postoperatively: chr5:173153463, chr6:64569557, chr12:61472036, chr21:20040011, and chr22:19192301. Table 2 also reports the nearest gene, the location of the CpG relative to the nearest gene, the mean preoperative percent methylation, and the mean change in percent methylation after surgery. The mean and 95% confidence interval of the methylation change observed after cardiac surgery for these significant CpGs are shown in Fig. 1. The mean change in DNA methylation and 95% confidence interval by type of cardiac surgery are shown in Fig. 2 for CpGs that have a statistically significant change after cardiac surgery. Notably, despite heterogeneity in the magnitude of change in DNA methylation by cardiac surgical procedure—likely due to lifestyle and genetic differences not controlled for in our study—the mean change in DNA methylation is in the same direction for all cardiac surgical procedures. We performed a permutation simulation to further support that the 5 CpGs with statistically significant changes in DNA methylation after surgery were not secondary to statistical noise within our analysis pipeline. In this permutation analysis, we determined the number of statistically significant (FDR < 0.05) sites after switching the preoperative and postoperative samples for each individual in a random selection of half of the patients. We then repeated this analysis 100 times. In this simulation, we found that 95 times out of 100 there are no statistically significant sites. In 4 times out of 100, there was 1 statistically significant site and 1 time out of 100 there were 3 statistically significant sites. Given our null hypothesis that we would find 5 sites by chance, we reject this null hypothesis with an empirical *p* value of < 0.01.

**Table 1:** Patient characteristics

Total patients	96
Age, mean ± SD	62.1 ± 13.1
Sex (M:F)	66: 30
Current smoker (number)	6
ASA class (3:4)	21: 75
Cardiopulmonary bypass time in minutes (mean ± SD)	142.8 ± 53.2
Anesthesia time in hours (mean ± SD)	7.7 ± 1.6
DHCA (number)	4
Isolated valve surgery (number)	35
Isolated CABG (number)	21
Isolated aortic surgery (number)	4
Myomectomy (number)	1
Combined surgery (number)	35

*SD*  standard deviation, *ASA*  American Society of Anesthesiologists, *DHCA*  deep hypothermic circulatory arrest, *CABG*  coronary artery bypass grafting. ASA class 3 denotes severe systemic disease that is not life-threatening and ASA class 4 denotes severe systemic disease that is a constant threat to life

**Table 2:** CpGs with a statistically significant change in DNA methylation after cardiac surgery

CpG	Nearest gene	Location	Pre-Op mean % methylation	Mean % methylation change	*P* value	FDR
chr5:173153463	BNIP1	Inside intron	60.84	4.79	1.36E-06	0.022
chr6:64569557	EYS	Inside intron	47.85	1.92	2.68E-06	0.022
chr12:61472036	LOC105369793	Downstream	72.25	−1.24	2.59E-06	0.022
chr21:20040011	LINC01683	Upstream	70.39	−0.61	2.35E-06	0.022
chr22:19192301	CLTCL1	Inside intron	62.02	4.67	2.04E-06	0.022

Five CpGs had a statistically significant change in DNA methylation after cardiac surgery (FDR < 0.05) in a paired analysis by individual that models methylation count data for all CpGs and controls for preoperative and postoperative cell-type heterogeneity

**Fig. 3:**
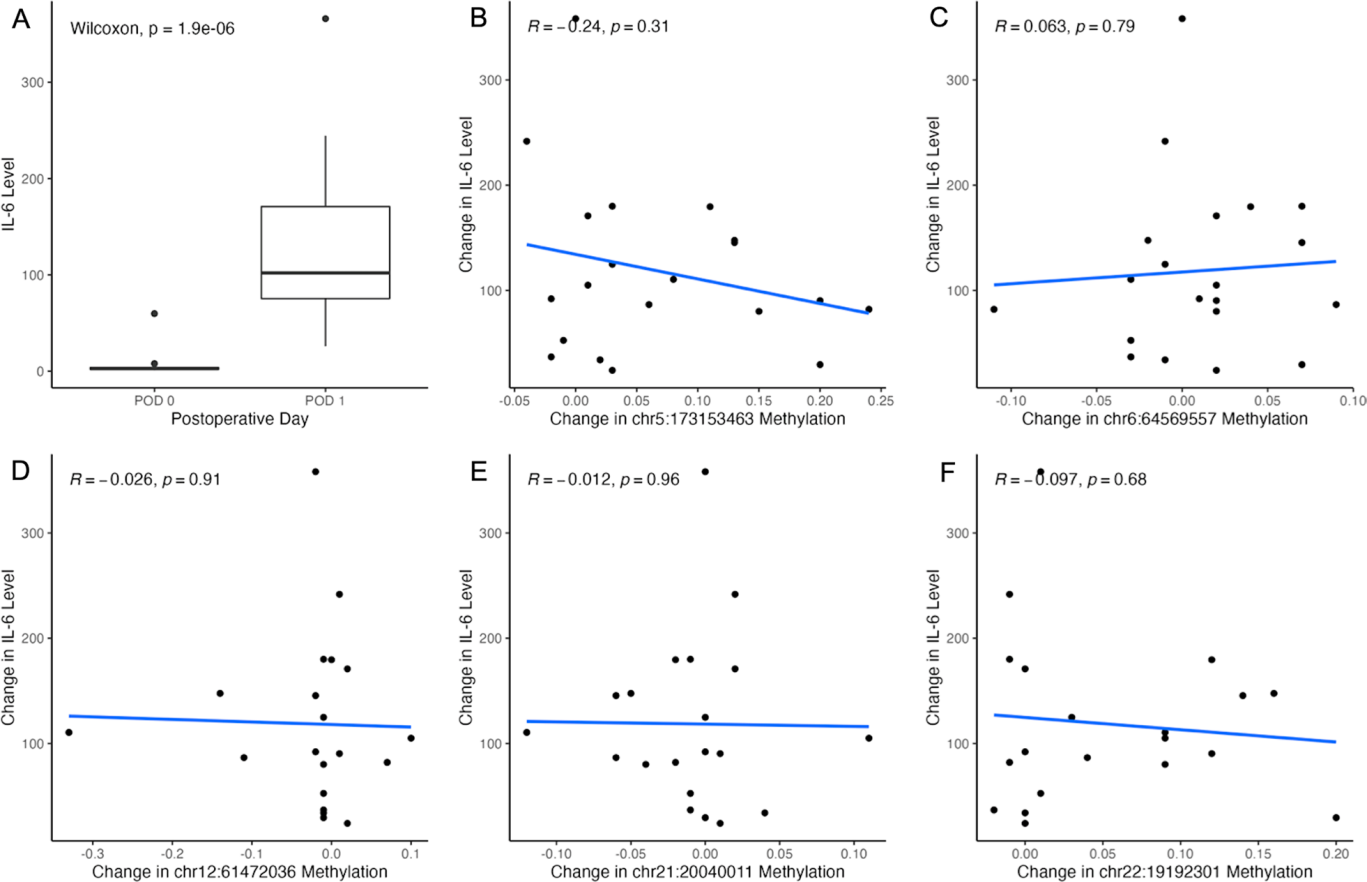
Significant DNA methylation changes after cardiac surgery are not a measure of the extent of Inflammation. Of the 96 patients in our study, 20 had preoperative and postoperative IL-6 levels available. Panel (**A**) shows that IL-6 levels increase significantly from preoperative to postoperative sampling, demonstrating increased inflammation after cardiac surgery. Panels (**B**) through (**F**) plot change in DNA methylation at 24 h by change in IL-6 level at 24 h for each CpG that has a statistically significant change in DNA methylation after cardiac surgery. Each point on these scatter plots represents one patient sample. A regression line and its accompanying R demonstrate a lack of linear relationship between change in DNA methylation after cardiac surgery and IL-6 levels

For a subset of 20 patients, preoperative and postoperative IL-6 levels were measured due to co-enrollment in another observational research study. These 20 patients were representative of the larger cohort, underwent the same type of surgeries, and received the same perioperative care. Figure 3A shows a significant change in IL-6 between the preoperative samples and samples collected 24 h later. IL-6 is a well-known inflammatory marker that correlates with the extent of tissue injury and inflammation after surgery [13]. Remarkably, changes in DNA methylation as a result of cardiac surgery are not simply a readout of the extent of inflammation, because we observe no significant association between the change in IL-6 levels and the change in DNA methylation (Fig. 3B–F) at 24 h after surgery.

We subsequently assessed the association of the 5 CpGs with statistically significant changes in DNA methylation with new-onset postoperative atrial fibrillation (POAF), a common complication after cardiac surgery. Of the 84 patients without a history of atrial fibrillation, 33 experienced POAF resulting in an incidence of 39%. Of the CpGs with statistically significant changes in DNA methylation after cardiac surgery, two CpGs (chr6:64569557 and chr22:19192301) showed changes in DNA methylation that were significantly associated with POAF (Table 3), with greater magnitude changes in DNA methylation being associated with POAF occurrence. Because we analyze the change in methylation for each patient (each patient serves as their own control), this statistical design controls for age and other covariates associated with POAF such as hypertension, diabetes, and chronic obstructive pulmonary disease. Additionally, the change in DNA methylation after surgery for chr6:64569557 and chr22:19192301 had a very low correlation and statistical association with age (Additional file 5: Figs. S4B and E, respectively), the most significant risk factor for POAF [12]. These findings further support methylation at these sites as independent factors associated with POAF.

**Table 3:** Association of CpGs that have statistically significant change in DNA methylation after cardiac surgery with postoperative atrial fibrillation (POAF)

CpG	Mean methylation change in patients with POAF	Mean methylation change in patients without POAF	*P* value
chr5:173153463	0.063	0.043	0.294
chr6:64569557	0.035	0.012	0.042
chr12:61472036	−0.021	−0.006	0.160
chr21:20040011	−0.007	−0.008	0.982
chr22:19192301	0.071	0.020	0.029

Of the 84 patients in this cohort without a history of atrial fibrillation, 33 experienced new-onset postoperative atrial fibrillation (POAF) after cardiac surgery for an incidence of 39%. For CpGs that have a statistically significant change in DNA methylation after cardiac surgery, the change in percent methylation in patients who experienced POAF was compared to the change in percent methylation in patients who did not experience POAF using the Wilcoxon rank-sum test. Notably, a greater magnitude change in DNA methylation is associated with POAF occurrence at chr6:64569557 and chr22:19192301, the two CpGs with statistically significant changes in DNA methylation associated with POAF

We next analyzed the methylation data using percent methylation rather than read count data with a linear mixed-effect model (Additional file 1: Table 1). Chr5:173153463 reached near statistical significance (FDR 0.07). Furthermore, chr5:173153463 and chr22:19192301 were two of the 5 most significant CpGs in the dataset analyzed with the linear mixed-effect model approach. Although this linear mixed-effect analysis using percent methylation data has less statistical power due to loss of granularity in the count data, this secondary analysis confirms that two of the 5 most significant CpGs are identified using two independent modeling approaches. Of note in our mixed-effect analysis, chr1:171597720 had a substantial mean decrease in DNA methylation of 13.5% after cardiac surgery. The nearest gene to chr1:171597720 is the myocilin opposite strand (MYOCOS). MYOCOS is a gene that is expressed in several tissues [14] including kidney, heart, lung, testis, brain, and adrenal gland. The function of this gene is unknown, and it is unclear why there is a significant decrease in methylation near this gene after surgery.

To assess the accuracy of in silico cell-type estimates, we compared preoperative and postoperative in silico cell-type estimates to fluorescence-activated cell sorting (FACS) measurements performed on fresh blood samples in a subset of 12 patients. Additional file 1: Table 2 shows that the in silico estimates are highly correlated with FACS measurements.

We next assessed the association of the statistically significant changes in DNA methylation independently with anesthesia time, cardiopulmonary bypass time, sex, and age. Although these were not covariates in our analysis to find statistically significant changes in DNA methylation after cardiac surgery, we were interested in how these patient characteristics and surgical factors are associated with changes in DNA methylation. Chr12:61470236 (Additional file 3: Fig. 2C) was found to be associated with cardiopulmonary bypass time (p = 0.031). Otherwise, the identified changes in DNA methylation after cardiac surgery are not significantly associated with anesthesia time (Additional file 2: Fig. 1), cardiopulmonary bypass time (Additional file 3: Fig. 2A, B, D, E), sex (Additional file 4: Fig. 3) or age (Additional file 5: Fig. 4). Taken together, these data suggest that the differentially methylated CpGs associated with POAF are signatures of an underlying epigenetic reprogramming that occurs in a subset of cardiac surgery patients and that these signatures are not downstream of known clinical parameters.

## Discussion

We report herein DNA methylation changes in whole blood samples induced by the physiologic stress of cardiac surgery on cardiopulmonary bypass. These DNA methylation changes reflect alterations in the immune response to the tissue injury that occurs during cardiac surgery. Importantly, two of the sites have postoperative changes in DNA methylation that are associated with POAF, a common and significant complication after cardiac surgery. Analysis such as this allows for the dissection of the immune responses to reveal processes associated with significant postoperative complications. This study introduces markers that could be used to track immune-mediated changes that are associated with clinically significant complications. Modulation of the immune response could yield a reduction in these complications which could be tracked with similar corrections in DNA methylation. This study also provides a framework to monitor epigenomic changes longitudinally in a large patient population and to control for cell-type variability in DNA methylation analyses.

Chr5:173153463, the CpG with the most statistically significant change in DNA methylation, is contained within an intron in BNIP1, a protein involved in vesicular transport into the endoplasmic reticulum and is associated with endoplasmic reticulum stress-associated apoptotic signaling [15]. BNIP3, another protein in the BNIP family, is a key regulator of apoptosis in ventricular myocytes during hypoxia [16]. Therefore, we posit that chr5:173153463 methylation in the immediate postoperative period may be involved in regulating cell death in response to hypoxia and ischemia–reperfusion injury after cardiac surgery. Chr22:19192301, another CpG with a statistically significant change in DNA methylation after cardiac surgery, is within an intron in CLTCL1, a protein of the polyhedral coat of coated pits and vesicles. CLTCL1, also called CHC22, is involved in the formation of insulin-responsive GLUT4 compartments in human muscle and adipocytes [17]. CLTCL1 methylation may be involved in upregulating glucose metabolism in response to the physiologic stress of cardiac surgery. Together, these sites are associated with modulating vesicular transport—whether regulating endoplasmic reticulum-related cell death or trafficking GLUT4 for glucose metabolism—which may incur significant perturbations in the physiologic response to surgery.

This study has many strengths which include its longitudinal design, the relatively large number of patients for a study of postoperative changes in DNA methylation, concomitant measurement of inflammatory marker IL-6 in a subset of patients, the availability of FACS data to support adequately controlling for cell-type heterogeneity and the association of statistically significant changes in DNA methylation with a clinically significant complication. In this study, proper control for cell-type heterogeneity is crucial to preventing false-positive associations due to differences in cell-type abundance between samples collected before versus after cardiac surgery. To this end, we calculated the Pearson correlation (or Spearman correlation if Shapiro–Wilk normality test *p* < 0.05) of in silico estimations of cell-type percent composition to measured cell-type composition from FACS in a subset of patients. The relatively high correlation of our in silico cell-type estimates with FACS data suggests sufficient control of cell-type heterogeneity using in silico cell-type estimates as covariates in our analysis. In addition, paired DNA methylation measurements and statistical analysis are an especially meaningful aspect of the experimental design as this controls for age, sex, and smoking status, characteristics known to be associated with altered DNA methylation. This research suggests that perioperative changes in DNA methylation and their association with common postoperative complications should be studied in larger cohorts. Such research could yield the discovery of further processes associated with unintended complications of the immune response and reveal potential targets for risk mitigation.

There are a few limitations to this study that should be noted. Although we sought to study changes in DNA methylation for patients that were representative of our university hospital system, the heterogeneity of cardiac surgical procedures may limit the generalizability of these findings to other cardiac surgical practices. Another limitation is that although reduced representation bisulfite sequencing (RRBS) measures DNA methylation throughout the genome, variation in restriction enzyme digestion can lead to low coverage or absent sites within individual samples which had to be excluded from our analysis, thereby resulting in fewer CpGs to analyze in the combined cohort. Future studies utilizing more extensive DNA methylation profiling (such as whole genome bisulfite sequencing) will likely yield additional methylation loci with significant changes after cardiac surgery. Although all blood products are leukocyte-reduced, transfusion can introduce a small amount of donor DNA [18] which could introduce noise in our dataset. To prevent false-positive detection from donor DNA, we specifically examined CpGs measured at ≥ 10X sequencing depth in the entire cohort and performed paired statistical testing. This strategy reduces the identification of spurious CpG changes in our cohort because we would have to see consistent methylation changes from the minuscule DNA contribution from random donors in all patients. Lastly, we did not obtain repeat DNA methylation measurements prior to discharge to determine whether the DNA methylation changes observed 24 h after surgery persisted throughout the postoperative period. Though it has been demonstrated that many postoperative changes in DNA methylation persist until postoperative days 4–7 [9], we did not verify the persistence of DNA methylation changes for sites identified in this study. Additionally, blood samples collected for RNA sequencing were not available to determine differential gene expression resulting from these changes in DNA methylation.

## Conclusions

This longitudinal profiling of DNA methylation in patients undergoing cardiac surgery identifies statistically significant postoperative changes in DNA methylation and demonstrates that such changes can be associated with significant postoperative complications. This research introduces a methodology that can uncover alterations in the epigenome that may reflect the susceptibility of the cardiovascular system to complications following surgery. This methodology may also be deployed to identify potential targets for risk mitigation of these significant complications. Future studies in larger cohorts will help us better understand the basic principles of the immune response to surgery at the gene level and identify potential therapeutic targets for the modulation of the immune response to reduce perioperative complications.

## Methods

### Patient enrollment and sample acquisition

After IRB approval and informed consent, adult patients undergoing non-emergent cardiac surgery on cardiopulmonary bypass at UCLA Ronald Reagan Medical Center were enrolled to participate in this prospective observational cohort study. Our aim of this study was to identify DNA methylation loci that undergo statistically significant changes after cardiac surgery. To accomplish this, we measured DNA methylation in preoperative and postoperative blood samples. We enrolled patients undergoing a variety of cardiac surgical procedures because this reflects the patient population in the university setting and the patients for which we would like to implement longitudinal epigenetic profiling. Patients responded to a questionnaire that recorded known methylation covariates including age, sex, and smoking status [19]. Prior to induction of anesthesia, 6 ml whole blood samples were obtained from the patient’s arterial line. For all patients, a postoperative 6 ml whole blood sample was obtained from the patient’s arterial line 24 h after the first sample. These whole blood samples were obtained in Becton Dickinson pink stoppered EDTA-containing vacuum blood collection tubes. For a subset of 12 patients, additional 6 ml preoperative and postoperative blood samples were available and were submitted for FACS to assess the accuracy of in silico cell-type estimates. Serum IL-6 levels were measured using preoperative and postoperative day 1 blood samples at UCLA Medical Center using routine clinical laboratory testing in a separate cohort of 20 patients undergoing cardiac surgery.

### DNA methylation data

Whole blood samples were immediately placed on ice and processed within 1 to 3 h. Genomic DNA was isolated from these whole blood samples (PureLink Genomic DNA Kit, K1820-01), digested with MspI (NEB, R0106S), and bisulfite converted. RRBS library preparation was adapted from prior studies [20–22]. RRBS libraries were prepared with 200 ng of DNA using the Illumina TruSeq kit (EpiTect Fast DNA Bisulfite Kit, Qiagen, 59,824), size selected for 330 bp with AMPure XP beads and underwent 100 bp single-end sequencing with Illumina HiSeq instruments. BS-Seeker2 [23] was used to align the reads to hg38, allowing for up to 10 mismatches. Methylation was called for CpGs and subsequently processed using methylKit [24] in R.

### Cell-type deconvolution

To account for the known confounding of cell-type composition on DNA methylation analysis in whole blood [25], we used a reference-based technique [20] that provides cell-type abundance estimates using DNA methylation sequencing data. Additionally, we performed FACS on preoperative and postoperative blood samples for 11 patients, which had additional blood samples available to assess the accuracy of reference-based cell-type deconvolution in our samples. FACS was performed by the UCLA Immunogenetics Clinical Laboratory to determine measurements for the percent composition of neutrophils, monocytes, B cells, CD4 + T cells, CD8 + T cells, and NK cells. These are the cell-type estimates typically used as covariates in accounting for cell-type heterogeneity [25] in whole blood samples. Notably, all six of these cell types are not available in complete blood cell counts used in the clinical setting.

### Statistical analysis

We filtered autosomal CpGs to require at least 10 reads in all preoperative and postoperative samples in all patients. Additionally, we removed CpGs located at SNPs with common allele frequency > 1% in humans (dbSNP build 150 common SNPs: http://hgdownload.cse.ucsc.edu/goldenpath/hg38/database/snp150Common.txt.gz).

All statistical tests were performed using R version 4.1.1. Using the DSS-general package [26] function DMLfit.multiFactor, we modeled CpG count data using a beta-binomial distribution and performed paired regression analysis on the count data with fixed effects for preoperative and postoperative cell-type estimates to determine CpGs undergoing significant methylation change after cardiac surgery. This paired analysis compares the change in methylation for each patient sample, holding age, smoking status, and sex constant in the statistical design. The statistical significance of postoperative status was called with the DSS-general function DMLtest.multiFactor. The abundance of cell types in whole blood, a known confounder of epigenetic studies [25], is also known to change after surgery [27] and thus these covariates were also controlled for in this paired sample analysis. Correction for multiple hypothesis testing was performed using the Benjamini–Hochberg procedure [28] with FDR < 0.05 considered statistically significant. As a conservative simplification of the proposed statistical model, we used a paired t test for our power calculation. A sample size of 96 gives adequate power (> 80%) to detect mean differences as small as 0.025 assuming an SD of 0.049, two-tailed, FDR-adjusted alpha = 0.0022, assumed correlation = 0.26). The assumed SD and correlation were observed averages across all markers in the data prior to analysis and the FDR-adjusted alpha was computed assuming an FDR rate of 5% and a true H_1_/false H_0_ rate of 5%.

For CpGs with statistically significant changes in DNA methylation after cardiac surgery, the association of the percent methylation change of these CpGs with POAF was analyzed with the Wilcoxon rank-sum test. Pearson or Spearman (Shapiro–Wilk normality test *p* < 0.05) correlations were calculated to determine the association between in silico cell-type estimates and cell-type abundance. For CpGs with statistically significant changes in DNA methylation after cardiac surgery, scatter plots were created using ggplot2 [29] in R to show the association of the change in percent methylation with each of the following patient data: IL-6 level, anesthesia time, cardiopulmonary bypass time, sex and age. For each scatter plot, the Pearson correlation, its associated p value and a regression line were also calculated using ggplot2 in R. The statistical significance of the difference between preoperative IL-6 levels compared to postoperative IL-6 levels was assessed with the Wilcoxon signed-rank test, a paired nonparametric statistical test.

Linear mixed-effect analysis was performed using the LME4 [30] package in R to create a multi-level linear regression model fitted for the percent methylation of each CpG with fixed effects for cell-type estimates, age, sex, and a random effect for each individual. Correction for multiple hypothesis testing was performed using the Benjamini–Hochberg procedure [28].

### Occurrence of postoperative atrial fibrillation

Patients were prospectively followed for occurrence of POAF with continuous electrocardiogram monitoring from intensive care unit admission until hospital discharge. Patients with a history of atrial fibrillation were excluded from the POAF occurrence analysis. POAF was defined as any occurrence of AF of at least 30-s duration diagnosed by a clinician. In this study, we defined POAF as a binary occurrence because we would like to capture any changes in DNA methylation that are associated with the development of POAF rather than episodes requiring treatment or that are sustained for longer durations.

## Supplementary Information

**Additional file 1. Supplemental Table 1. Linear Mixed Effects Analysis of DNA Methylation Change after Cardiac Surgery.** This multi-level linear regression model fitted the percent methylation of each CpG with fixed effects for cell type estimates, age, sex and a random effect for each individual. This table shows the five most significant CpGs in this analysis. **Supplemental Table 2. Comparison of Preoperative and Postoperative in silico Cell Type Estimates with Fluorescence Activated Cell Sorting (FACS) Measurements.** FACS was performed in a subset of 12 patients to evaluate the efficacy of in silico cell type estimates. Pearson correlation was used except when Shapiro-Wilk normality test *p*.

**Additional file 2**:** Fig. S1**. Statistically Significant Postoperative DNA Methylation Changes are not Associated with Anesthesia Time. Panels (A) through (E) plot change in DNA methylation by anesthesia time in hours for each CpG that has a statistically significant change in DNA methylation after cardiac surgery. Each point on these scatter plots represents one of the 96 patient samples. A regression line and its accompanying R demonstrate a lack of linear relationship between change in DNA methylation after cardiac surgery and anesthesia time.

**Additional file 3**:** Fig. S2**. Association of Significant Postoperative DNA Methylation Changes with Cardiopulmonary Bypass Time. Panels (A) through (E) plot change in DNA methylation by cardiopulmonary bypass time in hours for each CpG that has a statistically significant change in DNA methylation after cardiac surgery. Each point on these scatter plots represents one of the 96 patient samples. A regression line and its accompanying R demonstrate a lack of linear relationship between change in DNA methylation after cardiac surgery and cardiopulmonary bypass time for all CpGs except chr12:61470236.

**Additional file 4**:** Fig. S3**. Statistically Significant Postoperative DNA Methylation Changes are not Associated with Sex. Panels (A) through (E) show box plots of patient sex by change in DNA methylation for each CpG that has a statistically significant change in DNA methylation after cardiac surgery. The significant overlap of these distributions demonstrate a lack of significant association between change in DNA methylation after cardiac surgery and sex.

**Additional file 5**:** Fig. S4**. Statistically Significant Postoperative DNA Methylation Changes are not Associated with Age. Panels (A) through (E) plot change in DNA methylation by age for each CpG that has a statistically significant change in DNA methylation after cardiac surgery. Each point on these scatter plots represents one of the 96 patient samples. A regression line and its accompanying R demonstrate a lack of linear relationship between change in DNA methylation after cardiac surgery and age.

## Data Availability

The data generated and analyzed in this study can be found in the GEO repository with the following requisition number: GSE215937.
