# Supplementary material for: Longitudinal profiling in patients undergoing cardiac surgery reveals postoperative changes in DNA methylation
Source: Clin Epigenetics. 2022 Dec 30;14:195. doi: 10.1186/s13148-022-01414-4 (PMC9805211; doi:10.1186/s13148-022-01414-4)
Supplement: Supplementary file 1 — Additional file 1. Supplemental Table 1. Linear Mixed Effects Analysis of DNA Methylation Change after Cardiac Surgery. This multi-level linear regression model fitted the percent methylation of each CpG with fixed effects for cell type estimates, age, sex and a random effect for each individual. This table shows the five most significant CpGs in this analysis. Supplemental Table 2. Comparison of Preoperative and Postoperative in silico Cell Type Estimates with Fluorescence Activated Cell Sorting (FACS) Measurements. FACS was performed in a subset of 12 patients to evaluate the efficacy of in silico cell type estimates. Pearson correlation was used except when Shapiro-Wilk normality test p. [file 13148_2022_1414_MOESM1_ESM.docx]

| **CpG** | **Nearest Gene** | **Location** | **Pre-Op Mean % Methylation** | **Mean % Methylation Change** | **P-value** | **FDR** |
| --- | --- | --- | --- | --- | --- | --- |
| chr1:171597720 | MYOCOS | upstream | 27.2 | -13.5 | 1.05E-05 | 0.21 |
| chr4:92702202 | GRID2 | inside intron | 9.4 | 7.5 | 8.14E-05 | 0.57 |
| chr5:173153463 | BNIP1 | inside intron | 60.8 | 4.8 | 1.74E-06 | 0.07 |
| chr19:45381962 | PPP1R13L | inside intron | 93.9 | 0.9 | 2.85E-05 | 0.38 |
| chr22:19192301 | CLTCL1 | inside intron | 62.0 | 4.7 | 4.43E-05 | 0.45 |

**Supplemental Table 1. Linear Mixed Effects Analysis of DNA Methylation Change after Cardiac Surgery.** This multi-level linear regression model fitted the percent methylation of each CpG with fixed effects for cell type estimates, age, sex and a random effect for each individual. This table shows the five most significant CpGs in this analysis.

**Supplemental Table 2. Comparison of Preoperative and Postoperative *in silico* Cell Type Estimates with Fluorescence Activated Cell Sorting (FACS) Measurements.** FACS was performed in a subset of 12 patients to evaluate the efficacy of *in silico* cell type estimates. Pearson correlation was used except when Shapiro-Wilk normality test p < 0.05 where Spearman correlation was instead calculated (denoted by *).

| **Cell Type** | **Mean *in silico* Estimate** | **Mean FACS Estimate** | **Correlation** |
| --- | --- | --- | --- |
| **Preoperative** |  |  |  |
| Neutrophils | 0.62 | 0.59 | 0.98 |
| Monocytes | 0.09 | 0.06 | 0.89* |
| T-Helper/Inducer | 0.15 | 0.11 | 0.86 |
| T-Suppressor/ Cytotoxic | 0.07 | 0.06 | 0.58* |
| B- Lymphocytes | 0.03 | 0.03 | 0.51* |
| NK | 0.04 | 0.03 | 0.8* |
| **Postoperative** |  |  |  |
| Neutrophils | 0.85 | 0.80 | 0.63 |
| Monocytes | 0.09 | 0.07 | 0.53 |
| T-Helper/Inducer | 0.04 | 0.03 | 0.65 |
| T-Suppressor/ Cytotoxic | 0.01 | 0.01 | 0.37* |
| B- Lymphocytes | 0.00 | 0.01 | 0.15* |
| NK | 0.01 | 0.01 | 0.07* |

**Supplemental Table 2. Comparison of Preoperative and Postoperative *in silico* Cell Type Estimates with Fluorescence Activated Cell Sorting (FACS) Measurements.** FACS was performed in a subset of 12 patients to evaluate the efficacy of *in silico* cell type estimates. Pearson correlation was used except when Shapiro-Wilk normality test p < 0.05 where Spearman correlation was instead calculated (denoted by *).
