# Supplementary figures and images for: Longitudinal profiling in patients undergoing cardiac surgery reveals postoperative changes in DNA methylation
Source: Clin Epigenetics. 2022 Dec 30;14:195. doi: 10.1186/s13148-022-01414-4 (PMC9805211; doi:10.1186/s13148-022-01414-4)

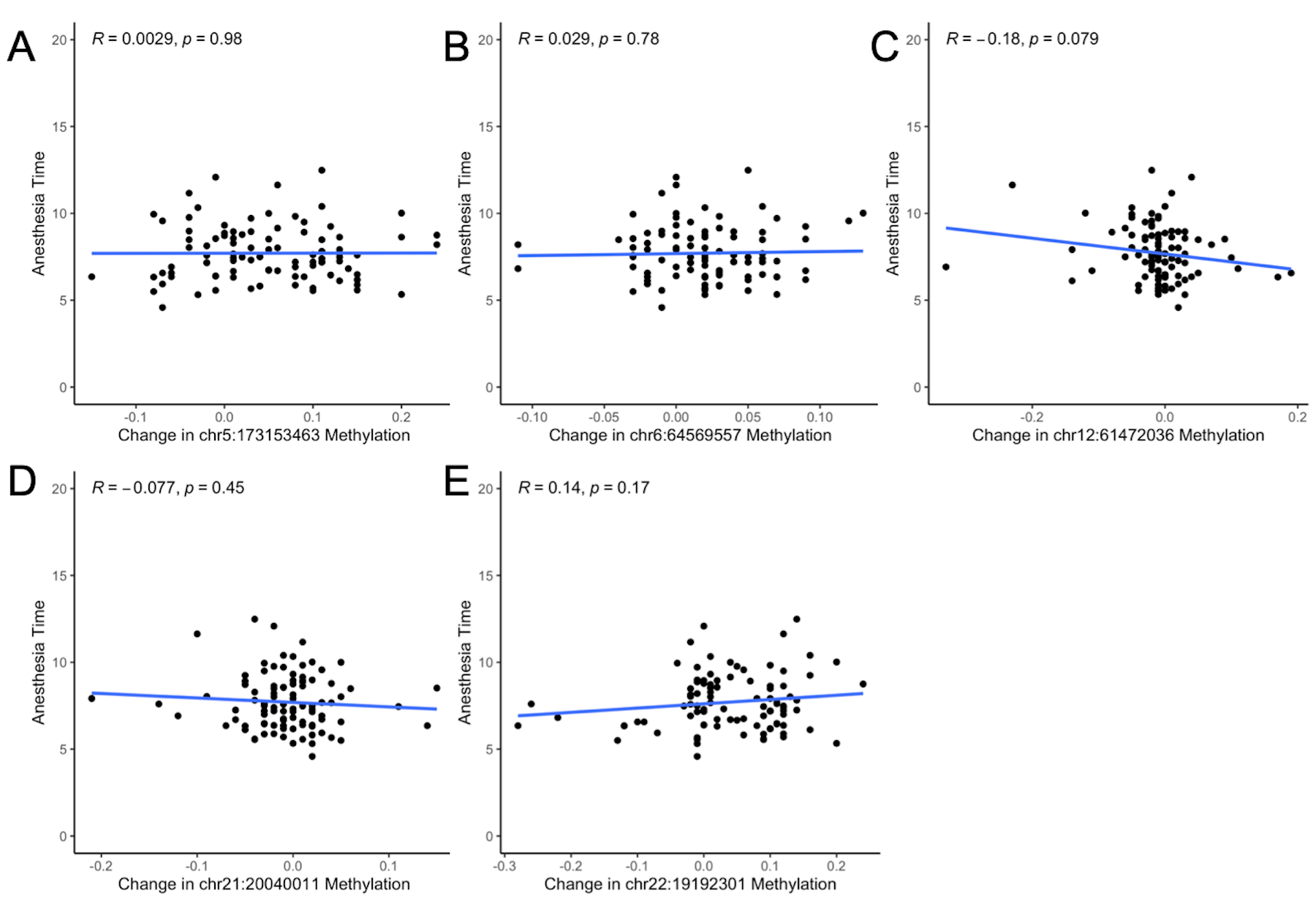

Supplement: Supplementary file 2 — Additional file 2: Fig. S1. Statistically Significant Postoperative DNA Methylation Changes are not Associated with Anesthesia Time. Panels (A) through (E) plot change in DNA methylation by anesthesia time in hours for each CpG that has a statistically significant change in DNA methylation after cardiac surgery. Each point on these scatter plots represents one of the 96 patient samples. A regression line and its accompanying R demonstrate a lack of linear relationship between change in DNA methylation after cardiac surgery and anesthesia time. [file 13148_2022_1414_MOESM2_ESM.tiff]

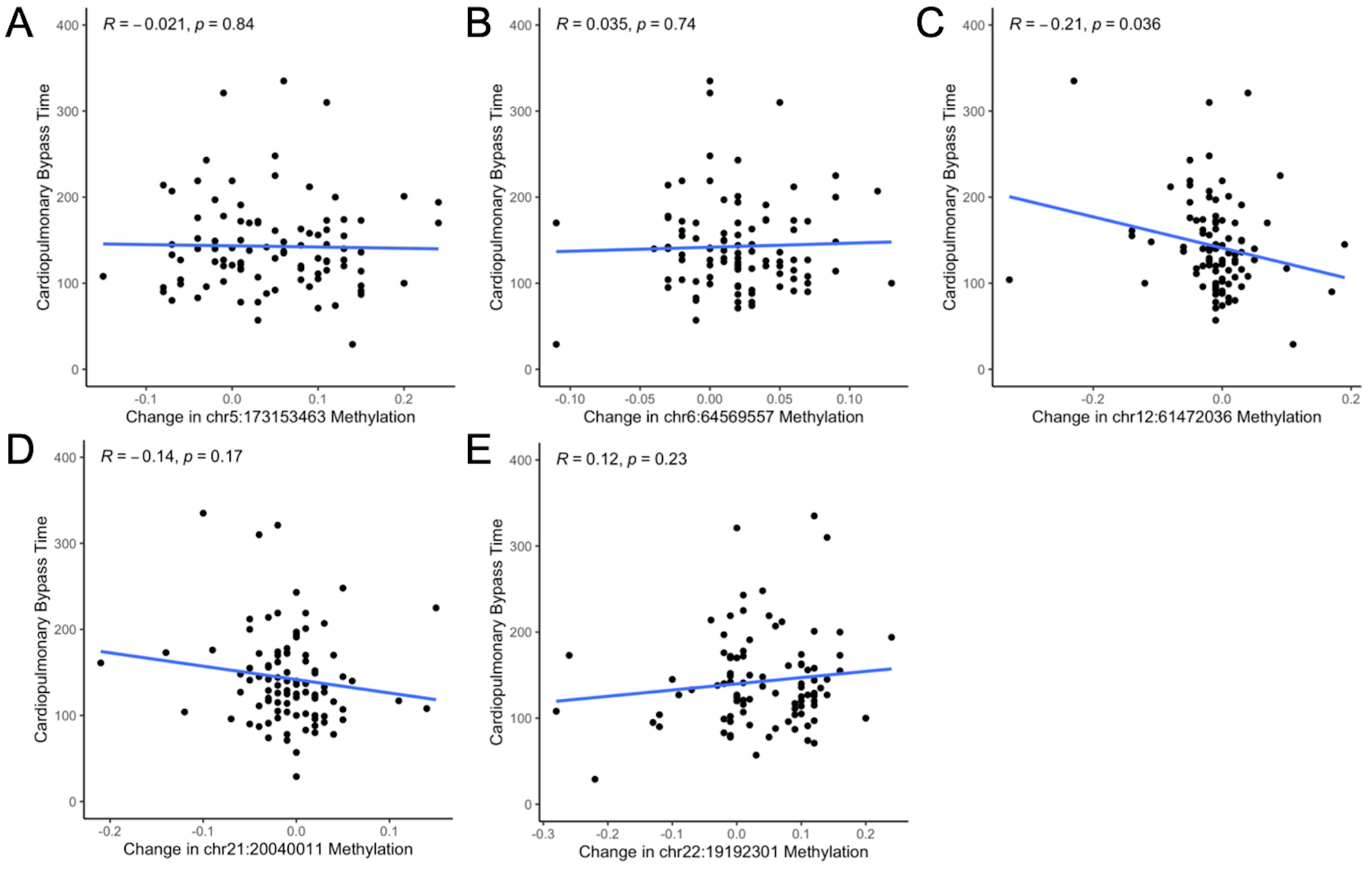

Supplement: Supplementary file 3 — Additional file 3: Fig. S2. Association of Significant Postoperative DNA Methylation Changes with Cardiopulmonary Bypass Time. Panels (A) through (E) plot change in DNA methylation by cardiopulmonary bypass time in hours for each CpG that has a statistically significant change in DNA methylation after cardiac surgery. Each point on these scatter plots represents one of the 96 patient samples. A regression line and its accompanying R demonstrate a lack of linear relationship between change in DNA methylation after cardiac surgery and cardiopulmonary bypass time for all CpGs except chr12:61470236. [file 13148_2022_1414_MOESM3_ESM.tiff]

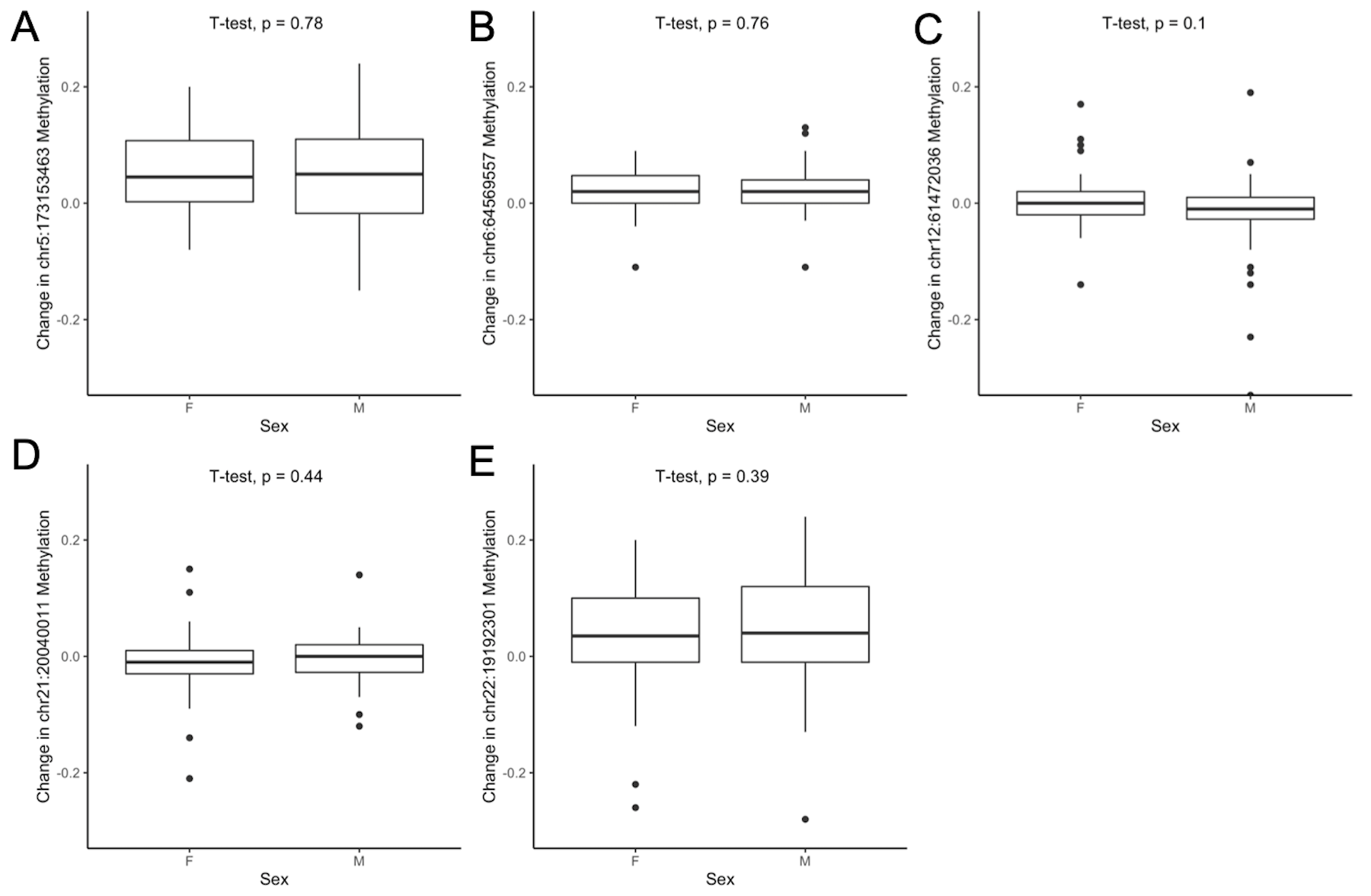

Supplement: Supplementary file 4 — Additional file 4: Fig. S3. Statistically Significant Postoperative DNA Methylation Changes are not Associated with Sex. Panels (A) through (E) show box plots of patient sex by change in DNA methylation for each CpG that has a statistically significant change in DNA methylation after cardiac surgery. The significant overlap of these distributions demonstrate a lack of significant association between change in DNA methylation after cardiac surgery and sex. [file 13148_2022_1414_MOESM4_ESM.tiff]

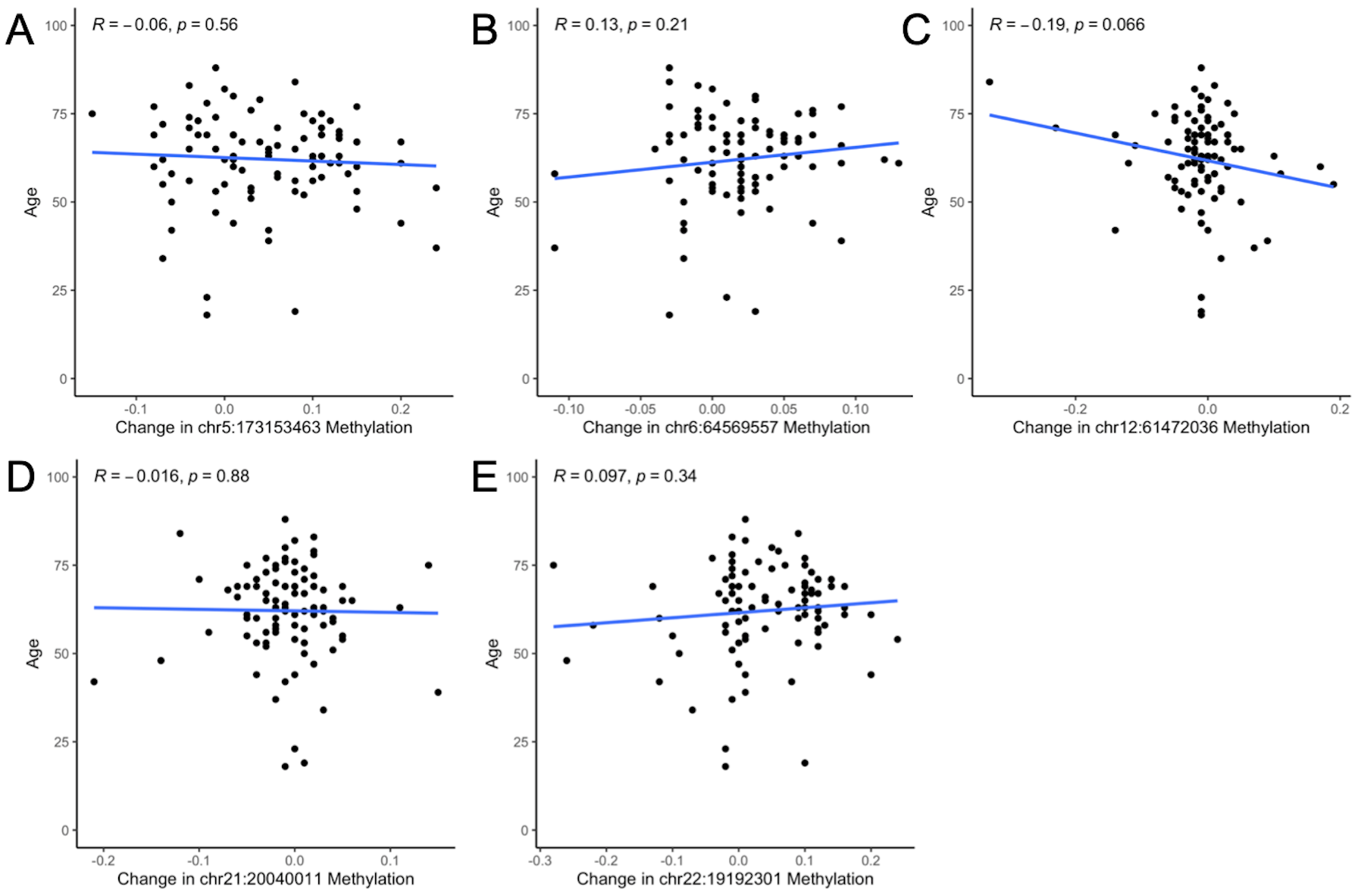

Supplement: Supplementary file 5 — Additional file 5: Fig. S4. Statistically Significant Postoperative DNA Methylation Changes are not Associated with Age. Panels (A) through (E) plot change in DNA methylation by age for each CpG that has a statistically significant change in DNA methylation after cardiac surgery. Each point on these scatter plots represents one of the 96 patient samples. A regression line and its accompanying R demonstrate a lack of linear relationship between change in DNA methylation after cardiac surgery and age. [file 13148_2022_1414_MOESM5_ESM.tiff]
